# Clustering patterns mirror the geographical distribution and genetic history of Lemnos and Lesvos sheep populations

**DOI:** 10.1371/journal.pone.0247787

**Published:** 2021-03-03

**Authors:** Antonios Kominakis, Eirini Tarsani, Ariadne L. Hager-Theodorides, Ioannis Mastranestasis, Ioannis Hadjigeorgiou

**Affiliations:** 1 Department of Animal Science, Agricultural University of Athens, Athens, Greece; 2 Breeder’s Association of the Lesvos sheep, Lesvos, Greece; National Cheng Kung University, TAIWAN

## Abstract

Elucidating the genetic variation and structure of Lemnos and Lesvos sheep is critical for maintaining local genetic diversity, ecosystem integrity and resilience of local food production of the two North Aegean islands. In the present study, we explored genetic diversity and differentiation as well as population structure of the Lemnos and Lesvos sheep. Furthermore, we sought to identify a small panel of markers with the highest discriminatory power to assign animals across islands. A total number of n = 424 (n = 307, Lemnos and n = 117, Lesvos) ewes, sampled from n = 24 herds dispersed at different geographic regions on the two islands, were genotyped with the 50K SNP array. Mean observed heterozygosity was higher (but not statistically significantly different) in Lesvos than in Lemnos population (0.384 vs. 0.377) while inbreeding levels were higher in Lemnos than Lesvos herds (0.065 vs. 0.031). Results of principal components along with that of admixture analysis and estimated genetic distances revealed genetic clusters corresponding to Lesvos and Lemnos origin and the existence of infrastructure within islands that were associated with geographical isolation and genetic history of the studied populations. In particular, genetic analyses highlighted three geographically isolated herds in Lemnos that are located at mountainous areas of the island and are characterized as representatives of the local sheep by historic data and reports. Admixture analysis also showed a shared genetic background between Lemnos and Lesvos sheep attributable to past gene flow. Little overall genetic differentiation was detected between the two island sheep populations, while 150 discriminatory SNPs could accurately assign animals to their origin. Present results are comparable with those reported in the worldwide sheep breeds, suggesting geography related genetic patterns across and within islands and the existence of the local Lemnos sheep.

## Introduction

Knowledge of the genetic variation within and between sheep populations is important for the identification of local genetic resources (i.e., individuals of local sheep breeds) to be maintained in animal genetics conservation efforts and the efficacy of applied selection programs. Preserving the genetic variability of livestock local genetic resources is important for ensuring food security under a changing climate and for sustaining productivity and resilience of agri-food chains. In Greece, sheep populations are reported to have been shaped by several factors such as geographical isolation, genetic drift, selection and crossbreeding [1]. Studies have already explored the genetic diversity and population structure of various sheep Greek breeds such as Frizarta [2], Chios [3], Karagouniko [3] and Lesvos [3,4].

The latter breed has been reported to cluster with other mountainous breeds in Greece [1]. It is placed on the center of an east–west geographical cline and is an intermediate between fat-tailed Asian and European sheep [5]. Using microsatellite markers, Mastranestasis et al. [4] estimated a heterozygote excess in the overall Lesvos sheep population as well as low genetic differentiation levels between herds attributable to high gene flow.

The Lesvos breed is a fat-tailed dairy sheep mainly located on the homonymous island of the North Aegean sea where about 260,000 purebred animals dispersed over 2,000 medium sized flocks (100–200 animals) are being kept under a semi-intensive production system (Ministry of Rural Development and Food, MRDF 2015 [6]). Apart from the Lesvos island, the breed is also found in the mainland and other Aegean islands where it is used to upgrade local sheep populations due to its pronounced milk productivity under harsh environmental conditions (dry heat areas and poor vegetation) [4].

Lemnos is another island of the North Aegean Sea neighbouring Lesvos, with a long tradition in small ruminants. Livestock farming on the island is dominated by dairy sheep totalling c.a. 60,000 ewes according to the EU’s Integrated Administration and Control System (IACS). Based on anecdotal reports, during the 1960s the local sheep population was extensively mated with the Lesvos breed. The Terra Lemnia project, aiming to evaluate the genetic resources of the island, conducted a survey among local livestock farmers regarding the characteristics of their farms. In this survey, 40 farmers were interviewed the majority of which characterized their sheep as crossbreds of the original local sheep mainly with Lesvos sheep and to a lesser extend with other Greek or foreign breeds. Nevertheless, pure-bred sheep belonging to the so called local Lemnos sheep were also reported at the mountainous Vigla region, at the northwestern part of the island. The survey data were supported by our own observations during visits to representative farms across the island, confirming high phenotypic variability within the Lemnos sheep population. This variability could be attributed either to extensive interbreeding or geographical isolation and local adaptation, with most distinctive that of the Vigla mountain (northwestern part of the island). In contrast to the Lesvos sheep breed that is largely purebred and relatively well characterized, the genetic makeup of the ‘modern’ Lemnos sheep remains largely unknown.

Sheep breeding is an important activity for both islands that shapes their ecosystem and contributes greatly to their agricultural, cultural and culinary heritage. In particular, milk from local ewes is used to produce cheeses, some of which are granted protected denomination of origin status e.g. the Ladotyri (Lesvos PDO), Kalathaki (Lemnos PDO) and Melichloro (Lemnos).

Driven from the importance of both sheep populations, in the present study we performed genetic analyses aiming at: (i) exploring the genetic makeup of Lemnos sheep and their relationship with the Lesvos breed, (ii) detecting population structure in the two populations and (iii) identifying a small panel of the most informative SNPs with high assignment power to predict island membership of animals. Current results are expected to shed more light in the evolutionary history of the two sheep populations and contribute to their better genetic management.

## Material and methods

### Ethics statement

All applicable ethical guidelines for the care and use of animals were followed and animal blood samples were collected by trained personnel under strict veterinary rules. All samples and data in our study were collected under the consent of the breeders. The protocol of the present study was approved by the Research Ethics and Ethics Committee of the Agricultural University of Athens (no 54/2020) according to the article 23 of law 4521/2018 of the Greek government.

### Data and quality control

A total number of n = 433 sheep blood samples were collected from 24 herds of the two islands. All animals were genotyped with the Illumina Ovine 50K SNP array by Neogen Europe Ltd (Ayr, UK). First, the accuracy of SNP genotyping was assessed and following the manufacturer’s recommendations, genotypes presented with a GenCall score greater than 0.30 were considered called. We then performed quality control (QC) both at the animal level and marker level. At the animal level, n = 9 samples were excluded due to call rate lower than 0.95 resulting in a total number of 424 samples in 24 herds in Lemnos (n = 307 in 18 herds) and Lesvos (n = 117 in 6 herds). At the marker level, n = 3,762 markers of the 43,647 autosomal SNPs were excluded due to: call rate lower than 0.95, minor allele frequency (MAF) lower than 0.05, deviation from Hardy–Weinberg equilibrium (HWE) using a Fisher exact test p-value<10^−4^ and linkage disequilibrium (LD) r^2^ levels (r^2^>0.50, window size: 50 SNPs, increment: 5 SNPs). Application of QC criteria at the marker level resulted in a total number of 39,885 autosomal SNPs retained for further analyses. QC was performed using the SNP & Variation Suite software (version 8.8.3).

### Genetic diversity and inbreeding

Measures of genetic diversity i.e. expected heterozygosity (*H*_*E*_) and observed heterozygosity (*H*_*O*_) were estimated per island and per herd within islands using the ‘gl.basic.stats’ function from the dartR R package [7]. The Brown and Forsythe’s test for homogeneity of variance was then applied to test for statistically significant differences of *Ho* between islands as well as differences between *He* and *Ho*, within islands. This test was performed using SAS ver9.0 (2002).

Individual inbreeding levels were assessed using two measures. First, the individual inbreeding coefficients (*f*_*is*_) were calculated using PLINK 1.9 [8] based on the observed versus expected number of homozygous genotypes. This inbreeding coefficient *f*_*is*_ is equivalent to Wright’s within-subpopulation fixation index *F*_*IS*_, with negative and positive values denoting heterozygote excess and deficit, respectively. Then, runs of homozygosity (ROH) per animal were detected using the following criteria: (i) the minimum number of SNPs included in a ROH was set at 25, (ii) the minimum length of a ROH was set at 1 Mb, (iii) one heterozygous genotype and no more than one missing SNP were allowed per ROH and (iv) the maximum distance between consecutive SNPs was set to 1 Mb. This analysis was performed using the ‘consecutiveRUNS.run’ function in the detectRUNS R package [9]. Next, individual genomic inbreeding coefficients based on ROH (*f*_*ROH*_) were calculated using the ‘Froh_inbreeding’ function in the detectRUNS R package [9]. Specifically, *f*_*ROH*_ were calculated from the sum of ROH lengths, divided by the total length of the autosomal genome covered by markers (2610 Mb), as proposed in [10]. Four *f*_*ROH*_ coefficients per animal were calculated using ROH of different length classes: (i) all ROH, (ii) 1 to 5 Mb, (iii) 5 to 20 Mb and (iv) >20 Mb. Finally, the degree of familial relationships i.e. shared ancestry between pairs of individuals sampled per herd was estimated using the $\hat{\pi }=P(IBD=2)+0.5P(IBD=1)$ summary statistic obtained by PLINK 1.9 [8], where *P*(*IBD* = 2) and *P*(*IBD* = 1) are the probabilities of two individuals carrying 2 or 1 SNP alleles identical by descent, respectively. $\hat{\pi }$ values can range from 0 for unrelated samples to 1 for duplicated samples (or twins). Intermediate values such as 0.0625, 0.125, 0.25 and 0.50 denote fourth (e.g. double cousins), third (e.g. first cousins), second (e.g. half-sibs) and first degree relatives (e.g. full sibs, parent-offspring).

### Detection of genetic structure

Detection of genetic structure was assessed using three different approaches. First, the genomic relationship matrix (GRM) between all pairs of individuals using genotypes of the 39,885 SNPs was calculated in PLINK 1.9 [8]. Elements of the GRM were then mapped to a color similarity matrix derived by Pearson moment correlations of the genomic relationships between pairs of individuals to produce a heat map. This heatmap was constructed via the Morpheus matrix visualization and analysis platform (https://software.broadinstitute.org/morpheus/).

Next, Principal Component Analyses (PCA) of animals’ genotypes was conducted to detect genetic clusters between and within island populations. Each time, the first 10 PCs were calculated using the ‘—pca’ command in PLINK 1.9 [8] and then pairwise plots of the main two PC were constructed to visually appraise clustering patterns in the data.

Finally, a variational Bayesian framework as implemented in the fastStructure [11] was employed to infer population structure using admixture analysis (AD). Variational Bayesian inference aims to repose the problem of inference as an optimization problem rather than a sampling problem and it is reported to deliver comparable results to ADMIXTURE in less runtime when using large numbers of genetic markers [11]. Here, K-values (i.e. the number of assumed ancestral populations) ranged from 2 to 8 and a five-fold CV procedure was performed to determine the optimal K-value presenting the lowest CV error. The default convergence criterion and prior were used as provided in the python script ‘structure.py’ (see: https://rajanil.github.io/fastStructure/). FastStructure results for each K cluster were graphically visualized using Distruct from CLUMPAK (http://clumpak.tau.ac.il/ [12]).

### Genetic differentiation and distances

Genetic differentiation both between and within islands were explored via pairwise estimations of fixation index (*F*_*ST*_) values between herds. Specifically, we used the ‘stamppFst’ function with a number of 1000 bootstraps and 0.95 confidence interval from the StAMPP [13] R package. Pairwise Nei’s genetic distances between herds were then calculated using the ‘stamppNeisD’ function in the StAMPP [13] R package and were graphically visualized using NeighborNet graphs constructed in SplitsTree5 [14].

### Discriminant analysis

Prediction of group membership of sheep samples of the two islands using limited numbers of SNPs with highest discriminatory power, followed. To this end, first we selected limited numbers (25 to 150) of strongly differentiated SNPs between the two islands defined as markers with highest *F*_*ST*_ values between the two islands. Next, we performed PCA on the various sets of differentiated markers as a dimensionality-reduction technique in attempts to obtain smaller sets of variables i.e. Principal Components that contain most of the variance in the original sets. Finally, Linear Discriminant Analysis (LDA) on the constructed PCs was applied using normal-theory methods assuming unequal variances, prior probabilities proportional to sample sizes and the CrossValidate (CV) option to obtain CV error-rate estimates. LDA was performed using the DISCRIM procedure in SAS ver 9.0 (2002). When the CV classification is specified, PROC DISCRIM classifies each observation in the data set using the discriminant function computed from the other observations in the data set, excluding the observation being classified. Thus, every data point is reclassified as if it were a new unknown observation. This provides a more conservative accuracy assessment. A total number of 16 cases arising from combinations of different numbers of SNPs and constructed PCs were examined during LDA. Results of LDA are presented as misclassification error rates per island and on average.

## Results

### Genetic diversity and inbreeding levels

Based on estimates of inter-locus diversity, average *H*_*O*_ was higher in Lesvos than in Lemnos population (0.384 vs. 0.377) (Table 1). Nevertheless, application of the Brown and Forsythe’s test for homogeneity of variance showed that this difference was not statistically significant (F = 192.12, df = 1, p<0.001). For Lemnos sheep, *H*_*O*_ was less than *H*_*E*_ (0.377 vs. 0.388, Brown and Forsythe’s test, F = 0.23, df = 1, p<0.64) while in the Lesvos sheep, *Ho* followed closely HW expectations (0.384 vs. 0.387, Brown and Forsythe’s test, F = 81.69, df = 1, p<0.001).

**Table 1:** Average observed heterozygosity (H_O_) and expected heterozygosity (H_E_) for the two islands.

Island		Mean	SD^a^	Min	Max
Lemnos	H_O_	0.377	0.110	0.036	0.608
	H_E_	0.388	0.111	0.042	0.500
Lesvos	H_O_	0.384	0.120	0	0.675
	H_E_	0.387	0.114	0	0.500

^a^Standard deviation.

**Fig 1:**
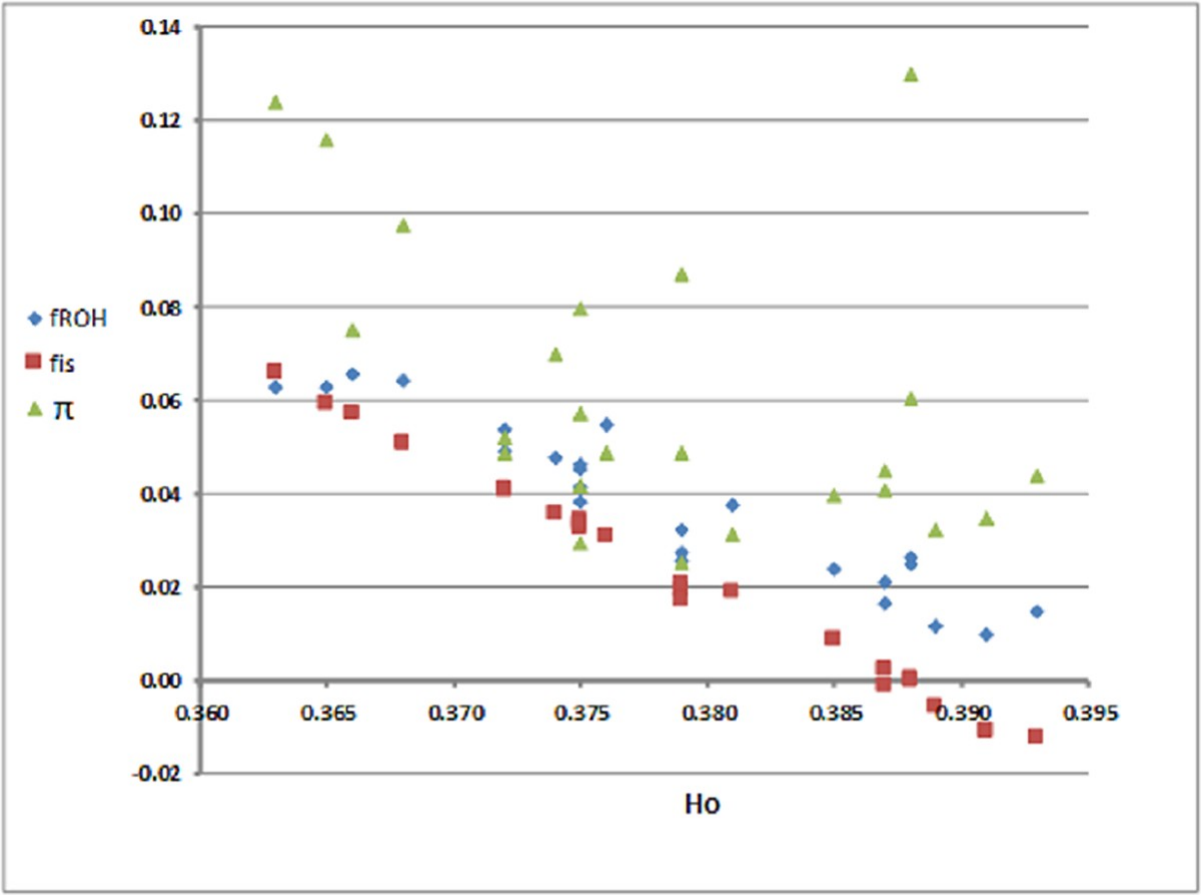
Scatter plot of observed heterozygozity (*H*_*O*_) against average inbreeding coefficients (*f*_*ROH*_, *fis*) and familial relationships ($\hat{\pi }$) per herd and island.

Levels of genetic diversity (*H*_*O*_) along with *f*_*is*_, *f*_*ROH*_ and $\hat{\pi }$ estimates across the herds of the two islands are shown in Table 2. *H*_*O*_ values varied more between Lemnos herds compared to Lesvos. Within Lemnos, *H*_*O*_ widely ranged from 0.363 (LEMN2) to 0.393 (LEMN16) with many herds displaying *H*_*O*_ values in the lower range (0.36–0.37). In contrast, average *H*_*O*_ of the Lesvos herds was always in the upper range (0.38–0.39). Estimates of average herd *f*_*is*_ ranged from -0.0125 (LEMN 16) to 0.066 (LEMN2) and ROH-based inbreeding coefficients (*f*_*ROH*_) from 0.015 (LEMN16) to 0.063 (LEMN2). Furthermore, overall average *f*_*is*_ estimates were higher in Lemnos (0.0284) than Lesvos (0.0066) and this was also the case for *f*_*ROH*_ (0.065 vs. 0.031, S1 Table). This trend was consistent across all ROH length classes and was strongly manifested for the long ROH lengths (>20 Mb) (0.119 vs. 0.076, S1 Table) that are indicative of recent parental relatedness. The highest inbreeding coefficient observed, *f*_*ROH*_ = 0.268, was in a Lemnos sample expected to be the result of a mating of close relatives (parent-offspring or full sibs). Estimates of shared ancestry ($\hat{\pi }$) across herds also ranged widely from 0.0253 (LESV2) to 0.130 (LEMN4). Of the 24 herds, three herds (LEMN1, LEMN4 and LEMN2) yielded average genomic relatedness greater than 0.10 consistent with the presence of multiple second and third order relatives (‘cryptic relatives’). Fig 1 presents a scatter plot of *H*_*O*_ against *f*_*ROH*_, *f*_*is*_ and $\hat{\pi }$ across herds. As this Figure depicts, there is a linear negative relationship, as derived by the Spearman correlation (*r*_*S*_), between *H*_*O*_ and *f*_*ROH*_ (r_S_ = -0.952, P<0.001) that was more apparent for the pair *H*_*O*_—*f*_*is*_ (*r*_*S*_ = -0.988, p<0.001) and less pronounced for pair *Ho*–$\hat{\pi }$ (*r*_*s*_ = -0.483, *p* = 0.0168). Note the close relationship between *f*_*is*_ and *f*_*ROH*_ estimates (*r*_*S*_ = 0.965, p<0.001).

**Table 2:** Average observed heterozygosity (*H*_*O*_), expected heterozygosity (*H*_*E*_), *f*_*ROH*_ and *f*_*is*_ within herds of Lemnos (LEMN) and Lesvos (LESV).

Herd	N	H_O_ (SD)	H_E_ (SD)	f_ROH_	SD_fROH_	f_is_	SD_fis_	$\hat{\pi }$
LEMN1	21	0.365 (0.18)	0.355 (0.15)	0.0628	0.052	0.0594	0.052	0.1159
LEMN2	20	0.363 (0.18)	0.353 (0.15)	0.0630	0.047	0.0661	0.049	0.1240
LEMN3	19	0.375 (0.17)	0.371 (0.14)	0.0380	0.041	0.0344	0.042	0.0573
LEMN4	7	0.388 (0.24)	0.354 (0.17)	0.0248	0.007	0.0004	0.009	0.1306
LEMN5	19	0.385 (0.17)	0.380 (0.13)	0.0238	0.014	0.0085	0.023	0.0395
LEMN6	16	0.366 (0.18)	0.365 (0.15)	0.0655	0.068	0.0573	0.069	0.0752
LEMN7	13	0.375 (0.18)	0.379 (0.14)	0.0415	0.046	0.0326	0.048	0.0295
LEMN8	20	0.387 (0.16)	0.382 (0.13)	0.0212	0.022	0.0024	0.027	0.0408
LEMN9	11	0.381 (0.18)	0.383 (0.14)	0.0375	0.032	0.0188	0.034	0.0311
LEMN10	13	0.375 (0.19)	0.367 (0.15)	0.0452	0.041	0.0334	0.043	0.0797
LEMN11	18	0.372 (0.17)	0.373 (0.14)	0.0491	0.029	0.0406	0.032	0.0487
LEMN12	20	0.376 (0.16)	0.375 (0.14)	0.0546	0.048	0.0307	0.049	0.0486
LEMN13	21	0.374 (0.17)	0.369 (0.14)	0.0478	0.036	0.0356	0.038	0.0700
LEMN14	21	0.375 (0.16)	0.379 (0.13)	0.0463	0.049	0.0335	0.070	0.0419
LEMN15	17	0.388 (0.17)	0.378 (0.14)	0.0262	0.011	0.0000	0.025	0.0602
LEMN16	22	0.393 (0.16)	0.385 (0.13)	0.0146	0.014	-0.0125	0.026	0.0437
LEMN17	20	0.372 (0.16)	0.373 (0.14)	0.0538	0.046	0.0409	0.047	0.0518
LEMN18	9	0.368 (0.21)	0.360 (0.16)	0.0642	0.032	0.0509	0.034	0.0975
LESV1	19	0.379 (0.17)	0.374 (0.14)	0.0273	0.025	0.0192	0.030	0.0488
LESV2	19	0.379 (0.16)	0.381 (0.13)	0.0254	0.022	0.0172	0.023	0.0253
LESV3	19	0.387 (0.16)	0.380 (0.13)	0.0163	0.013	-0.0013	0.018	0.0450
LESV4	19	0.389 (0.16)	0.383 (0.13)	0.0116	0.005	-0.0056	0.012	0.0323
LESV5	21	0.379 (0.17)	0.368 (0.14)	0.0323	0.040	0.0208	0.042	0.0871
LESV6	20	0.391 (0.16)	0.385 (0.13)	0.0098	0.005	-0.0108	0.017	0.0346

The sample size (N) and standard deviation (SD) are also provided.

### Genetic structure

**Fig 2:**
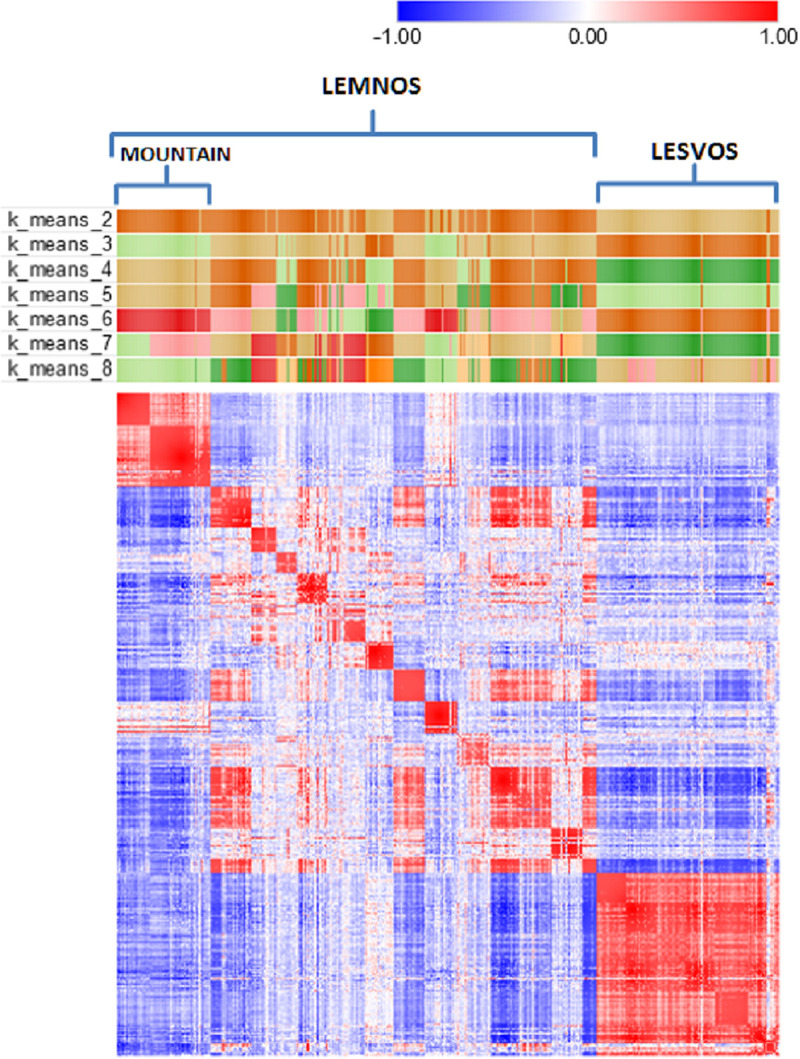
Heatmap of the genomic relationship matrix of 424 sheep from Lemnos and Lesvos along with *k*-means clustering (k = 2–8). The heatmap presents a color similarity matrix derived by Pearson moment correlations of the genomic relationships between pairs of individuals where red and blue colors indicate high (+1) and low (-1) genetic relationships, respectively. The presence of high intensity off-diagonal clusters indicates population structure. Small sized red colored squares on the diagonal denote high genomic relationships of animals at herd level. Large sized red colored squares at the upper left and lower right show the high genomic relationships of animals of the first three herds of Lemnos (mountain) and the six herds of Lesvos, respectively, that were grouped as separated clusters during *k*-means clustering.

Fig 2 shows a heatmap of the animals’ genetic relationships along with *k*-means clustering in attempts to detect patterns in the data according to genomic relatedness and/or geography. Visual inspection of the heatmap revealed a certain degree of clustering of animals at the herd level (depicted as small sized red colored squares on the diagonal) and most interestingly the formation of two off-diagonal clusters depicted as large sized red colored squares, one at the upper left formed by animals of three Lemnos herds (LEMN1, LEMN2 and LEMN3) and another at the lower right with animals of the six Lesvos herds. Note that the three Lemnos herds that were represented as a separate group during *k*-means clustering are topographically isolated herds, located in the northwestern part of the island on the Vigla mountain and are considered representatives of the local Lemnos sheep.

**Fig 3:**
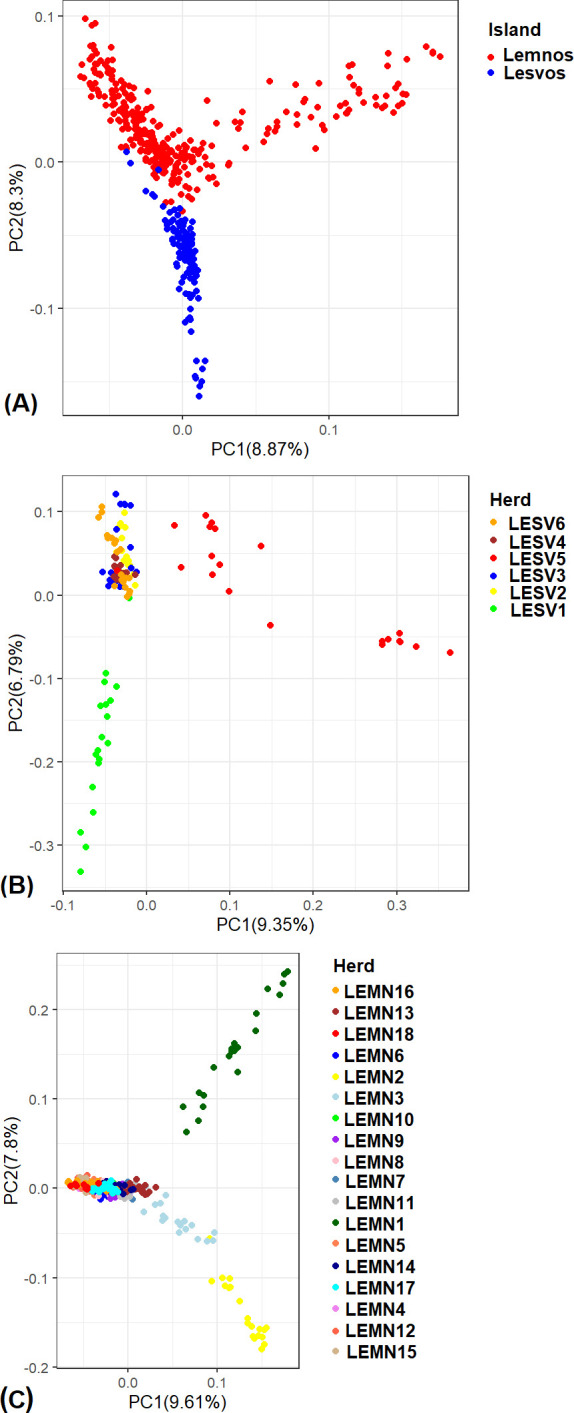
Two-dimensional plots of the first two principal component (PC) axes of genetic variation (labeled PC1 and PC2) of sheep genotypes per island (A), for Lesvos (B) and Lemnos (C). The percentage of variance each PC axis explained is also provided. Each point represents an individual genotype and points are colored by island (top) and herds within islands (middle and bottom). Plots were constructed using the ggplot2 R package [15].

Two-dimensional plots of the first two PCs derived from PCA on the 424 genotypes and 39,885 markers across and within islands are shown in Fig 3. The first top principal component (PC1) for all genotypes (Fig 3A) accounted for 8.9% of variance in the genetic data and was associated with variability of Lemnos genotypes (red color). In the same plot, the second top principal component (PC2, 8.3% of the genetic variability) was associated with variability of Lesvos genotypes (blue color) and separated most Lesvos from Lemnos samples. PC2 also demonstrated the closeness of Lesvos and Lemnos sheep populations as some (n = 10) Lesvos samples had PC2 values similar to Lemnos sheep (see center of the plot). PCA plot of Lesvos genotypes in the two-dimensional space (Fig 3B) revealed genetic infrastructure within the island, with herds LESV1 (green color) and LESV5 (red color) appearing as separated genetic groups from the remaining herds. Genetic infrastructure was also detected within Lemnos in the two-dimensional space. Here, there were three distinct clusters detected corresponding to herds LEMN1 (green), LEMN2 (yellow) and LEMN3 (light grey) (Fig 3C). Overall, PCA plots increased detection resolution by disclosing previously undiscovered genetic infrastructure within islands. In all the PCA plots the explanatory power of the first two PC axes was low (explaining about 16% - 18% of variation in the genetic data) implying that there could be more undetectable genetic structure in the data, not identifiable via two dimensional plots.

**Fig 4:**
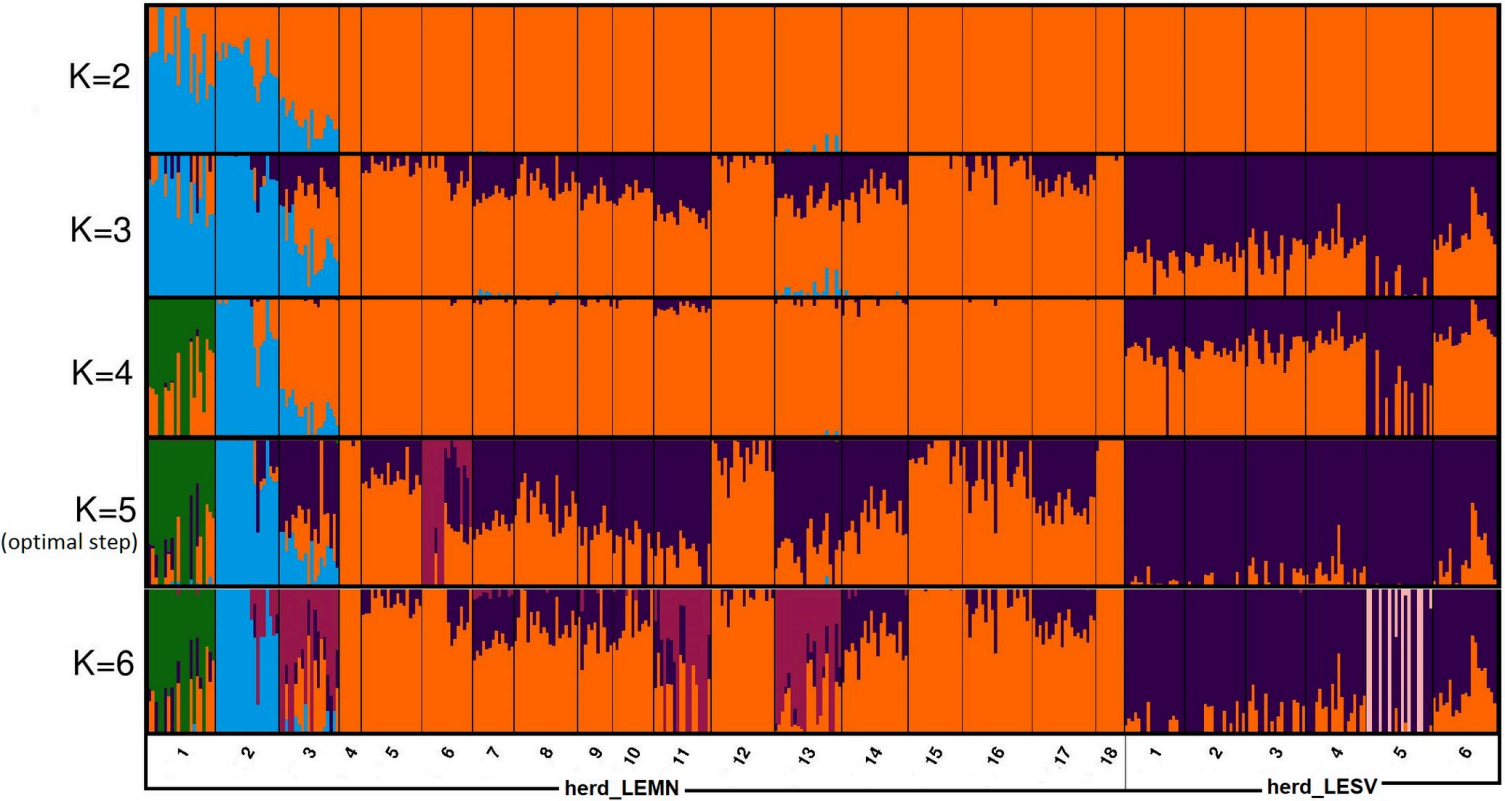
Genetic clusters as ancestry proportions of Lemnos and Lesvos sheep revealed by admixture analysis in fastStructure [11]. The x-axis represents individuals sorted according to herd_ID within islands. Each color represents a population and each individual is represented by a vertical line partitioned into colored segments whose lengths represent the admixture proportions from K ancestral populations. Plots were constructed in CLUMPAK (http://clumpak.tau.ac.il/, [12]).

Detection of genetic clusters as ancestry proportions via AD is shown in Fig 4. With the number of clusters (K) set at 2 (CV error = 0.6098) only one distinct cluster was generated that included animals of herds LEMN1, LEMN2 and LEMN3 (light blue color). This cluster was also persistent at K = 3 (CV error = 0.6086) when another cluster corresponding to Lesvos sheep (dark blue color) emerged. At K = 4 (CV error = 0.6163), animals of herds LEMN1, LEMN2 and LEMN3 split-off into two groups suggesting different ancestry genetic backgrounds for animals of herd LEMN1 vs. herds LEMN2 and LEMN3.

Note that at K = 3 and 4 Lesvos genotypes were represented as an admixed population. At the optimal step (K = 5) i.e. lowest CV error (0.6020), there were four major clusters generated, formed by herd LEMN1 (green color), herds LEMN2 and LEMN3 (light blue color), herd LEMN6 (purple color) and the Lesvos breed-specific cluster (dark blue color).

AD results largely concord with outcomes of *k*-means clustering of the GRM heatmap and PCA confirming genetic distinctness of the three Lemnos herds and the Lesvos sheep. Nevertheless, AD disclosed an additional finding. Many of the Lemnos sheep were represented as admixed individuals with variable and occasionally sizeable degree of Lesvos sheep ancestry (dark blue color, Fig 4). Such admixture patterns are typical for interbreds and fully comply with historical reports and phenotypic appearance of the animals.

### Genetic differentiation and genetic distances

The two sheep populations showed little overall genetic differentiation as derived by the overall *F*_*ST*_ estimate (0.009). S2 Table shows pairwise *F*_*ST*_ estimates between herds that ranged from 0.014 (LESV4—LESV6) to 0.096 (LEMN2-LEMN4). Results of *F*_*ST*_ were also confirmed by estimates of Nei’s genetic distances between herds, with lowest distances (0.025) attained for herds LESV4-LESV6 and highest (0.085) for herds LEMN2-LEMN4 (S3 Table).

**Fig 5:**
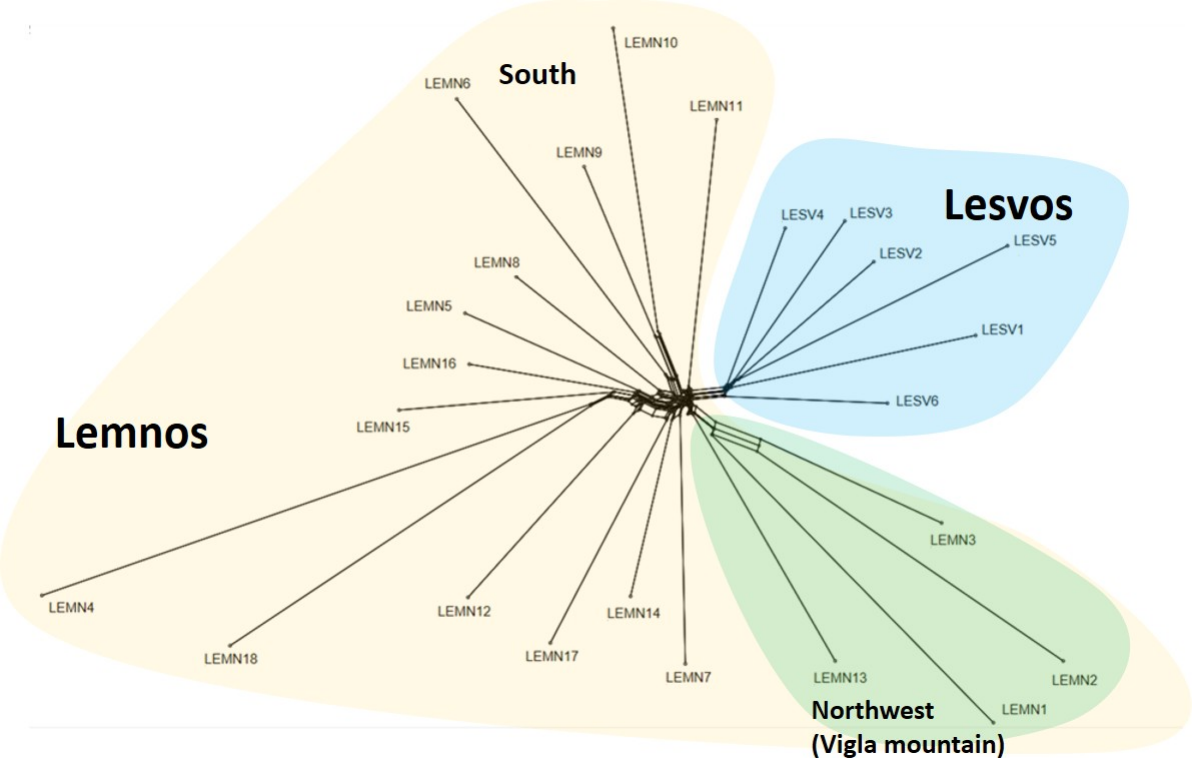
Neighbornet graph of Lesvos and Lemnos sheep based on estimated pairwise Nei’s genetic distances between herds. Yellow background color denotes herds of Lemnos and blue background color herds of Lesvos. For more details, see text. The graph was constructed in SplitsTree5 [14].

In accordance with GRM, *k*-means clustering and PC analyses findings, Neighbor-network phylogenetic analysis of Nei’s genetic distances revealed two separate branches (Fig 5), one corresponding to Lesvos (blue background color) and the other to Lemnos herds (yellowish background color). Within Lemnos, three branches formed, one comprised by herds LEMN1, LEMN2, LEMN3 and LEMN13, located at the northwest part of the island (green background color), a second with herds LEMN6, LEMN9 and LEMN10, located at the southern part of the island and a third including herds showing a wider geographical distribution. Note that long terminal distances of herds LEMN4 and LEMN18 reflect the increase of Nei’ distances resulting from small samples (see S3 Table).

### Prediction of group membership

Results of LDA across the various scenarios examined are presented in Table 3. Using only 25 markers resulted in an average misclassification error rate as high as 4.25% mainly due to high error rates (10.26%) for the Lesvos samples. The use of 50 markers improved the performance of the classification criterion resulting in average error rates of 1.42%. A SNP panel comprising 100 strongly differentiated markers further improved the performance of the classification criterion resulting in lower average error rates (0.24%). Notably, employment of panel of the 150 most differentiated SNPs was associated with nil misclassification error rates (Table 3).

**Table 3:** Cross-validation misclassification error rates of discriminant analysis of strongly differentiated SNPs.

	F_ST_		Misclassification error rates		
Number of markers	Mean (SD^a^)	Number of Principal Components	Lemnos	Lesvos	Average
25	0.159 (0.034)	1	0.0195	0.1026	0.0425
		2	0.0195	0.1026	0.0425
		5	0.0228	0.1026	0.0448
		10	0.0195	0.1026	0.0425
50	0.140 (0.030)	1	0.0065	0.0342	0.0142
		2	0.0065	0.0342	0.0142
		5	0.0065	0.0342	0.0142
		10	0.0065	0.0513	0.0189
100	0.1247 (0.027)	1	0.0000	0.0085	0.0024
		2	0.0000	0.0085	0.0024
		5	0.0033	0.0427	0.0142
		10	0.0098	0.0256	0.0142
150	0.115 (0.025)	1	0.0	0.0	0.0
		2	0.0	0.0	0.0
		5	0.0065	0.0000	0.0047
		10	0.0195	0.0000	0.0142

^a^Standard deviation.

## Discussion

### Genetic diversity and inbreeding

Both sheep populations studied herein maintained sufficient genetic variation as inferred by *H*_*O*_ with values in the range of 0.36 to 0.39. These estimates are comparable or higher to other southern and western European (0.22–0.38) [16] sheep breeds, are higher than the Oxford sheep breed (0.35) in the United States [17] and comparable (Lemnos) or higher (Lesvos) than other Greek breeds such as Chios, Kymi and Lesvos [5].

Of the two populations, Lemnos herds displayed less heterozygosity than expected under random mating. The most plausible cause for the heterozygote deficit is the mating of close relatives i.e. inbreeding [18]. The linear negative correlation between observed heterozygosity and inbreeding levels (Fig 1) and the increased inbreeding coefficients estimated for Lemnos herds (Table 1), suggests that heterozygote deficiency in the Lemnos sheep can be attributed to higher incidence of matings between relatives. In line with these findings, a linear inverse relationship between genetic diversity and inbreeding has also been established in other studies of Balkan and European sheep breeds [5].

Aside from inbreeding, heterozygote deficits can also be interpreted as evidence of the Wahlund effect [19] that occurs when differentiated subpopulations are sampled together (cryptic population structure). This may have also been the case here, as the Lemnos sample consists of several subsamples containing admixtures of individuals from different subpopulations in proportions that were variable from one subsample to the other. However, given that the herds exhibited little genetic differentiation (*F*_*ST*_ values in the range from 0.01 to 0.09), the Wahlund effect is an unlikely explanation for the observed negative relationship between inbreeding and *H*_*O*_. Furthermore, in contrast to the positive correlation between interlocus *f*_*st*_ and *f*_*is*_ that is theoretically expected as a result of the Wahlund effect [20,21], here a low negative correlation (r = -0.063, p<0.001) was estimated (results not shown).

Finally, cryptic relatedness i.e. non-random sampling of members from a limited number of families may also cause deviations from Hardy-Weinberg proportions [22–24]. Cryptic relatedness could be hypothesized here, as estimates of shared ancestry ($\hat{\pi }$) across herds (see Table 1 and Fig 1) indicate that in some cases, samples were collected from family members with third or even second degrees of consanguinity ($\hat{\pi }$~0.125–0.25). However, progeny sampled from a small number of progenitors are expected to exhibit a slight heterozygote excess relative to HW proportions [22–24] in contrast to the apparent heterozygosity deficit here. The latter finding allows us to conclusively reject non-random sampling from limited number of reproductive groups as a source of heterozygote deficiency leaving inbreeding as the only plausible explanation.

As in other studies (e.g. [2]), current results suggest that the use of dense genetic marker information is valuable for depicting the existing inbreeding levels, particularly when pedigree data are not available, as in our case. Both *f*_*is*_ and *f*_*ROH*_ estimates delivered comparable results that largely coincide. A close relationship between the two measures (*f*_*is*_ and *f*_*ROH*_) has also been observed in other Greek breeds, e.g. Frizarta [2], or foreign sheep breeds [25]. Of the two measures, *f*_*ROH*_ estimates present the best choice as they give a clearer insight on past and recent inbreeding history of animals [26,27]. Particularly, the highest *f*_*ROH*_ estimates for the long ROH lengths measuring >20 Mb obtained herein that reflect recent inbreeding history, underscore the need for efficient genetic management of the sheep populations to avoid close relative matings, especially for the Lemnos sheep. Recent inbreeding has been identified as a major determinant of the current genetic diversity and effective population size of many Greek sheep breeds (e.g. Frizarta [2], Boutsiko, Karagouniko and Chios [28]).

### Genetic differentiation and discriminatory power of differentiated markers

Average *F*_*ST*_ values estimated herein indicate little genetic differentiation between Lesvos and Lemnos sheep populations. To further support this finding, AD results show that the genetic makeup of most Lemnos sheep examined here has largely been shaped by the Lesvos sheep. This genetic closeness of the two populations can most likely be attributed to past gene flow from Lesvos to Lemnos island as suggested by historic data, local reports and phenotypic appearance.

Another interesting finding obtained here relates to the discriminatory power of differentiated markers in efforts to devise a tool for tracing the origin of local sheep and possibly their products. The idea here was first to identify limited numbers of strongly differentiated markers across islands, then to combine most of their genetic information into small number of variables (one or two PCs), and finally to derive a PC-based discriminatory criterion that could be used to accurately predict animals’ island membership. Present results have shown that the aforementioned approach could be practically implemented via development of a custom-made 150 SNP array that could be made available at an affordable price for large scale analyses. This SNP chip array could be used to discriminate Lesvos sheep from Lemnos sheep as long as no further gene flow occurs between the two islands. Although the discriminatory power of the 150 SNP panel under consideration was assessed using a conservative approach such as CV classification, its discriminatory performance further warrants to be tested using ‘true’ validation procedures by separating calibration and test data sets and a larger number of animals.

Michailidou et al. [28] have also demonstrated that a specialized marker panel including a total number of 3,802 SNPs can be applied for traceability purposes (identification of purebreds and crossbreds) of other indigenous Greek sheep breeds. Similar to the present study, Dimauro et al. [29] also showed that a small number of 108 SNPs were capable to discriminate 21 sheep breeds. Discriminatory SNP panels for breed assignment have also been identified for goats [30] and cattle [31].

### Geography related genetic structure and implications

Geographical isolation confounded with genetic origin as well as past interbreeding appeared to be key determinants of genetic structure patterns detected in the present study. The most important implication of the genetic patterns detected herein was the identification of three specific clusters within Lemnos that correspond to equal number of topographically isolated herds located at the rough terrain of the Vigla mountain. According to local farmers interviewed, animals of these herds are morphologically reminiscent of the local Lemnos sheep that historically prevailed across the island some decades ago. More recently, particularly in the lowland areas of Lemnos, the original local sheep were extensively interbred with other indigenous sheep from neighboring islands or other exotic breeds. The pure-bred Lemnos local sheep population was progressively restricted to the mountainous areas and declined in numbers, resulting in only about 800–900 animals remaining today mainly at the Vigla mountain. Due to their high adaptability and resilience, the owners of these remaining herds do not interbreed their animals with other indigenous or exotic sheep breeds. In contrast, interbreeding is still common practice in the lowland areas.

Given the critical census size and the importance of these animals in terms of maintenance of local genetic diversity and ecosystem integrity, immediate conservation interventions should be undertaken as isolation, together with small population size could result in the decline and ultimately extinction of this population [32,33].

## Conclusions

The use of genomic data information revealed that Lesvos and Lemnos sheep populations are clustered according to geographical location and/or genetic origin. Within Lemnos, genetic structure patterns were associated either with geographical isolation of local genetic resources or interbreeding with indigenous or exotic sheep breeds. Practical on-ground guidance and broader management actions should be undertaken to ensure the conservation of the ‘rediscovered’ Lemnos sheep.

## Supporting information

S1 Table Calculated f_ROH_ per length class and island. (DOCX)

S2 Table Pairwise F_ST_ estimates among herds of Lesvos and Lemnos. (XLSX)

S3 Table Nei’s genetic distances between herds of Lesvos and Lemnos. (XLSX)
