# Supplementary material for: Clustering patterns mirror the geographical distribution and genetic history of Lemnos and Lesvos sheep populations
Source: PLoS One. 2021 Mar 3;16(3):e0247787. doi: 10.1371/journal.pone.0247787 (PMC7928510; doi:10.1371/journal.pone.0247787)
Supplement: S1 Table — (DOCX) [file pone.0247787.s001.docx]

S1 Table. Calculated f_ROH_ per length class and island.

| ROH | **Island** | | | | | |
| --- | --- | --- | --- | --- | --- | --- |
|  | Lesvos | | | Lemnos | | |
|  |  | **F_ROH_** | |  | **F_ROH_** | |
| length  class (Mb) | n | Average | Range | n | Average | Range |
| 1 to 5 | 117 | 0.025 | 0.001- 0.171 | 307 | 0.057 | 0.001-0.268 |
| 5 to 20 | 80 | 0.058 | 0.007- 0.171 | 264 | 0.082 | 0.005-0.268 |
| >20 | 14 | 0.076 | 0.029-0.171 | 90 | 0.119 | 0.026-0.268 |
| All | 117 | 0.031 | 0.001-0.171 | 307 | 0.065 | 0.001-0.268 |
